# Long-Term Region-Specific Mitochondrial Functionality Changes in Both Cerebral Hemispheres after fMCAo Model of Ischemic Stroke

**DOI:** 10.3390/antiox13040416

**Published:** 2024-03-29

**Authors:** Ksenija Lūcija Bahire, Reinis Maļuhins, Fiona Bello, Jolanta Upīte, Aleksandrs Makarovs, Baiba Jansone

**Affiliations:** Department of Pharmacology, Faculty of Medicine, University of Latvia, LV-1586 Riga, Latvia; maluhins.reinis@inbox.lv (R.M.); fionabello23@gmail.com (F.B.); jolanta.upite@lu.lv (J.U.); aleksandrs.makarovs@lu.lv (A.M.)

**Keywords:** middle cerebral artery occlusion, ischemia/reperfusion injury, cerebral hemispheres, high-resolution fluorespirometry, oxygen consumption, reactive oxygen species, mitochondrial damage

## Abstract

Cerebral ischemia/reperfusion (I/R) refers to a secondary brain injury that results in mitochondrial dysfunction of variable extent, leading to neuronal cell damage. The impact of this process has mainly been studied in the short term, from the early hours up to one week after blood flow reperfusion, and in the ischemic hemisphere only. The focus of this study was to assess the long-term impacts of I/R on mitochondrial functionality using high-resolution fluorespirometry to evaluate state-dependent activities in both ischemic (ipsilateral) and non-ischemic (contralateral) hemispheres of male mice 60, 90, 120, and 180 days after I/R caused by 60-min-long filament-induced middle cerebral artery occlusion (fMCAo). Our results indicate that in cortical tissues, succinate-supported oxygen flux (Complex I&II OXPHOS state) and H_2_O_2_ production (Complex II LEAK state) were significantly decreased in the fMCAo (stroke) group ipsilateral hemisphere compared to measurements in the contralateral hemisphere 60 and 90 days after stroke. In hippocampal tissues, during the Complex I&II ET state, mitochondrial respiration was generally lower in the ipsilateral compared to the contralateral hemisphere 90 days following stroke. An aging-dependent impact on mitochondria oxygen consumption following I/R injury was observed 180 days after surgery, wherein Complex I&II activities were lowest in both hemispheres. The obtained results highlight the importance of long-term studies in the field of ischemic stroke, particularly when evaluating mitochondrial bioenergetics in specific brain regions within and between separately affected cerebral hemispheres.

## 1. Introduction

The energy demands of various cells in the brain are among the highest of all body organs, with a metabolism that is in continuous need of substrates for mitochondrial oxygen production. Healthy brain cells require a continuous supply of oxygen and ATP that is produced from glucose in mitochondria, an essential organelle involved in oxidative phosphorylation, mitochondrial permeability transition pore (mPTP)-mediated calcium homeostasis, reactive oxygen species (ROS) control, and programmed cell death pathways such as apoptosis and necrosis [1,2,3]. Considering the pathophysiology of ischemic stroke, mitochondrial damage is a key factor in and contributes to the continued progression of ischemic injury [4,5,6].

An ischemic stroke is a debilitating cerebrovascular event characterized by sudden occlusion of blood flow to a brain region, resulting in morphological changes to neuronal and glial cells, and often leads to neurodegeneration and neurological deficits [6,7,8]. In the ischemic core, neurons undergo morphological changes where cell bodies and axons disappear, while in the ischemic penumbra, neurons experience the disintegration of endoplasmic ribosomes and Nissl bodies [9]. Mitochondrial dysfunction is the most immediate response to oxygen and glucose deprivation following ischemia and is closely associated with ROS-mediated oxidative stress as a result of homeostatic imbalance between ROS production and biological antioxidant systems [6,10]. During an acute brain ischemia event, oxidative metabolism and energy production in the cerebral cortex are found to be severely damaged [11]. When blood flow to ischemic regions is restored, “secondary energy failure” because of reperfusion injury becomes evident—after a seemingly complete recovery of aerobic ATP production in the first phase of reperfusion, mitochondria function later fails as accumulated succinate is reoxidized and drives ROS generation via reverse electron transport at Complex I [12,13]. Complex IV activity then becomes dramatically reduced, which leads to cytochrome c release and subsequent cell death [14]. 

It has been demonstrated that enzyme activities related to energy transduction (citrate synthase, malate dehydrogenase, succinate dehydrogenase, etc.) exhibit different behavior in cortical tissue mitochondria during aging and complete cerebral ischemia, as well as 1 to 96 h after post-ischemic recovery [15]. Previous reports indicated partial recovery of mitochondrial respiratory capacity in the first hour of blood flow reperfusion following a 2 h long middle cerebral artery occlusion with secondary deterioration 2 to 4 h after in the penumbra and neocortical regions; full recovery of mitochondrial respiratory activity was observed in samples from the cortex but not the striatum regions [16,17,18]. 

Ischemic stroke can also cause significant alterations in the morphology and connectivity of hippocampal neurons, including a reduction in the number and density of dendritic spines, an essential structure in synaptic plasticity and learning [5,19]. It has been shown that a 15 min long ischemia/reperfusion (I/R) event inhibits mitochondrial translation machinery, leading to impacts on mitochondrial protein synthesis up to 24 h after reperfusion in rat hippocampus [20]. Hippocampal tissue consists of an ischemic-vulnerable CA1 region and ischemia-resistant regions (CA2, CA3, and CA4 and the dentate gyrus) that have more efficient energy metabolism due to mechanisms of glucose delivery and the regulation of key glycolytic enzymes [21]. Studies have shown that mitochondrial content parameters, as well as pro-fission and pro-fusion parameters, were significantly higher in ischemia-resistant regions (CA2/3 compared to the CA1 region) 24 to 96 h after transient global I/R in gerbils; in ischemia-vulnerable regions, greater neuronal loss and decreased enzymatic activity of citrate synthase was observed [22]. 

In our recent study on mitochondrial functioning 2 h after ischemic stroke, lower mitochondrial oxygen consumption and higher ROS production were observed in the stroke group’s ipsilateral hemisphere compared to the stroke group’s contralateral hemisphere and compared to the sham group’s ipsilateral hemisphere data [23]. In previous study designs, the contralateral hemisphere was tested as an internal control; increases in mitochondrial proton leak, maximal respiration, and spare capacity in contralateral hemisphere tissues were evident when compared with naïve animal tissue, indicating cerebral arteries are also affected at sites distant from direct ischemic stress [24]. It was also observed that mitochondrial depolarization significantly increased Ca^2+^ sparks in the ipsilateral, but not in the contralateral, hemisphere in middle cerebral arteries during a 90 min long transient MCAo (tMCAo) in rats, with ipsilateral arterial oxygen consumption rates greater than in the contralateral 48 h after reperfusion [24]. Other tMCAo rat model studies, with a 60 min long occlusion, experienced significant blood–brain barrier (BBB) damage 30 days after the tMCAO event—mostly in the ipsilateral striatum and motor cortex regions, with widespread microvascular alterations found in ipsilateral and contralateral brain hemispheres, suggesting persistent and/or continued BBB damage following chronic ischemia [25]. 

Given these observed changes to mitochondrial bioenergetics shortly after I/R damage, it is hypothesized that hemispheric differences would persist several months after transient ischemic stroke in mice. Therefore, the aim of this study was to investigate a mouse model time course of region-specific mitochondrial oxygen consumption and ROS emission changes after transient ischemic stroke in both cerebral hemispheres. This study represents, to the best of our knowledge, the first comprehensive mouse model evaluation of cortical and hippocampal mitochondrial functionality after ischemia/reperfusion during distinct respiratory states (e.g., phosphorylated and non-phosphorylated states, maximal electron transport) over time (60, 90, 120, and 180 days) and between ischemic and non-ischemic hemispheres. The use of an established mouse model of 60 min filament-induced middle cerebral artery occlusion followed by mitochondrial analysis with cutting-edge high-resolution respirometry (HRR) sheds further light on the intricate processes of mitochondrial adaptation and long-term dysfunction in response to ischemic injury with observed regional variations in mitochondrial bioenergetics and respiratory efficiency.

## 2. Materials and Methods

### 2.1. Animals 

In this study, male C57Bl/6NHsd mice 24 to 26 g in weight and aged 12 to 13 weeks were procured from Charles River Laboratories (Sulzfeld, Germany). Study mice were housed at the University of Latvia Faculty of Medicine Animal Care Facility within polysulfone cages (capacity of 5–6 mice; width of 395 mm, depth of 346 mm, and height of 213 mm) on individually ventilated stainless steel racks (Green Line IVC, GRM900 cages, Techniplast, Buguggiate, Italy). The mice were maintained in a controlled environment with a temperature of 23 °C, humidity of 50–60%, and a 12 h day/night cycle from 08:00 until 20:00. Each cage contained autoclaved aspen (*Populus tremula* L.) wood bedding (1031004), aspen wood wool (1034005), and aspen blocks (1023005) from LBS-biotech (London, UK) as well as a polycarbonate tunnel (K3487) and crawl ball (K3329) from Techniplast (Varesa, Italy) for environmental enrichment. All mice had free access to filtered tap water and pelleted chow containing protein (19.2%), fat (4.1%), fiber (6.1%), and ash (5.9%) (1324, Altromin, Lage, Germany). Mice were habituated to the animal facility for 7 days before the start of experimentation.

### 2.2. Ethics Statement

In compliance with EU Directive 2010/63/EU, ARRIVE guidelines, and local animal research protection regulations—and with approval from the Animal Ethics Committee of the Food and Veterinary Service (Riga, Latvia; Permit No. 100)—this experimental study was designed to enrich animals during life, minimize distress during experiments, and ultimately reduce total animal sacrifice. Humane handling actions were made during pre-operative care, surgical manipulation, post-operative care, and endpoint decisions.

### 2.3. Chemicals

Required chemical groupings included surgical analgesics/anesthetics, components for respiration medium (MiR05), modified substrate-uncoupler-inhibitor titration_(SUIT) protocol reagents, and ROS (H_2_O_2_) measurement reagents. The surgical analgesics, carprofen (Carprofelican, 50 mg/mL) and buprenorphine (Bupredine Multidose, 0.3 mg/mL, Produlab Pharma B.V., Raamsdonksveer, The Netherlands), as well as surgical anesthetic, isoflurane, were obtained from Produlab Pharma B.V. (Raamsdonksveer, The Netherlands). For mitochondrial incubation when using the Oroboros O2k respirometer, MiR05 respiration medium (pH 7.1 at 37 °C) reagents were sourced from Sigma-Aldrich (St. Louis, MO, USA) and prepared according to manufacturer composition (https://wiki.oroboros.at/images/c/c8/MiPNet22.10_MiR05-Kit.pdf accessed on 5 November 2023). Reagents and inhibitors used during the modified SUIT-008 protocol and H_2_O_2_ measurements were also purchased from Sigma-Aldrich (St. Louis, MO, USA), excluding adenosine diphosphate (ADP), which was purchased from Merck (Darmstadt, Germany). Reagents for SUIT included malic, pyruvic, and succinic acids; cytochrome c; ADP; glycerophosphate; carbonyl cyanide 3-chlorophenylhydrazone (CCCP); rotenone; and antimycin A. Reagents for H_2_O_2_ measurements included pentetic (DTPA), succinic, and pyruvic acids; superoxide dismutase (SOD); horseradish peroxidase; ADP; rotenone; antimycin A; and Amplex UltraRed (AmR). AmR is a light-sensitive chemical that can undergo photooxidation—it was light-protected during storage and used to maintain chemical integrity [26]. 

### 2.4. Intraluminal Filament Induced Middle Cerebral Artery Occlusion Model (fMCAo)

Induction of cerebral ischemia in mice was achieved through the occlusion of the middle cerebral artery (MCAo) using an intraluminal filament [27]. Pre-operative analgesia was administered thirty minutes prior to surgery via intraperitoneal (i.p.) injection of 5 mg/kg carprofen and subcutaneous (s.c.) injection of 0.1 mg/kg buprenorphine. A facemask anesthesia system (16-2025, Higlandmedical, Temecula, CA, USA) was used to administer 4.5% isoflurane, maintained at 1.5% to 2.0%, in a mixture of O_2_ (0.3 L/min) and N_2_O (0.7 L/min). During surgery, mouse temperature (37.0 °C ± 0.5 °C) was maintained using heating pads with real-time feedback response (RT-0501, Kent Scientific Corp, Torrington, CT, USA) while heart rate and oxygen saturation were monitored (PhysioSuite, Kent Scientific Corp., Torrington, CT, USA). Deep anesthesia was confirmed through the lack of response to a toe pinch. Fur and skin in the surgical area were shaved and disinfected prior to incision. Blood flow in the left middle cerebral artery region, as determined by projection on the skull bone surface, was monitored using a Laser-Doppler flowmetry fiber (moorVMS-LDF, Moor Instruments, Axminster, UK) placed in a small incision between the left eye and ear.

Occlusion lasting 60 min in the left middle cerebral artery induced blood flow decreases 20–55% of the magnitude of pre-occlusion blood flow values [27]. During occlusion, mice were placed in a recovery box (V1200DT, MediHeat, Weymouth, UK) with temperature maintained at 28.5 °C ± 0.5 °C. Following occlusion, mice were re-anesthetized, and flowmetry filament was removed; Sham group mice experienced identical handling. However, filaments were shallowly placed and did not induce decreases in blood flow [28]. Further fMCAo surgery procedure details are found in our previous work [23].

Post-surgical care was comprehensive and lasted 1 week. Pre- and post-surgical weight were recorded, and mice were actively monitored for pain and/or hypothermia. Immediately post-surgery, mice received 0.5 mL of saline injected s.c. to the withers and then placed for 2 h in a recovery box (Recovery Chamber-V1200, Peco Services Ltd., Cumbria, UK) set to 28.5 °C ± 0.5 °C. Segregated based on stroke or sham occlusion, mice were housed up to 5 in standard cages containing nesting material and a semisphere house (Mouse House, Techniplast, Varese, Italy) for environmental enrichment. Ambient room temperature in the mouse housing room was maintained at 24 °C ± 0.5 °C. To decrease mortality, adequate food and water intake was facilitated for the first five days by providing fresh daily Petri dishes containing jelly food (D59494, Sniff Spezialdiäten GmbH, D-59494 Soest, Germany) and hydrogel (ClearH_2_O Inc., Westbrook, ME, USA) [29]. Further electrolyte supplementation was provided via daily s.c. injection of 20% glucose solution (0.5 mL) and Ringer’s Lactate solution (0.5 mL). Post-surgical analgesics included 0.1 mg/kg of s.c. buprenorphine administered every 8–12 h for 2 days and 5 mg/kg i.p. carprofen administered every 24 h for 4 days. Inclusive to the whole study, the survival rate of mice was 90%.

To validate the fMCAo model, infarct volume was quantified 24 h after fMCAo in cresyl violet–stained 30 μm thick coronal sections, as previously described [23,30]. Sections were obtained using a freezing cryotome (CM1850, Leica Biosystems, Deer Park, IL, USA), scanning was conducted using whole slide Pannoramic MIDI II scanner (3DHISTECH Ltd., Budapest, Hungary) with a 20× microscope objective, and infarction areas were delineated and quantified using ImageJ software 1.54f (NIH). Infarct volume was calculated as the ratio of the infarction area to the whole hemisphere area.

### 2.5. Brain Tissue Homogenate Preparation

Mice were euthanized by cervical dislocation within the same 30 min time frame on days 60, 90, 120, and 180 after reperfusion. Following sacrifice and within 2 min, skulls were gently opened with scissors, whole brains were extracted and placed on an ice-cold stainless-steel plate, and blood was removed with an ice-cold MiR05 rinse. While excluding the corpus callosum, cortical and hippocampal tissues from left and right hemispheres were dissected, placed separately into ice-cold MiR05, and washed several times with MiR05 to remove remaining blood. Excess liquid was removed by transferring to filter paper (Whatman™, Maidstone, UK). Sample wet-weights were then determined by weighing on an analytical balance (±0.001 g, Boeco, Hamburg, Germany). 

Dissected samples—cortical tissues and hippocampal tissues from left and right hemispheres—were individually homogenized for 10 pestle strokes at medium speed within first pre-cooled loose fit and then pre-cooled tight fit glass Dounce homogenizers (KIMBLE Dounce tissue grinder, DWK Life Sciences GmbH, Rockwood, TN, USA) in a calculated volume (see equation below) of ice-cold MiR05 [31]. Freshly prepared homogenates were immediately transferred to labeled 2.0 mL Eppendorf tubes and stored on ice until analysis with the Oroboros O2k respirometer, 2 to 4 h from the time of isolation. For each sample, calculated homogenization volumes of MiR05 were determined such that sample mass concentration equaled 20 mg/mL:

$$Volume\;of\;MiR05\;(\mathrm{mL})=\frac{mass\;of\;sample\;\left(g\right)}{concentration\;of\;sample\;\left(\frac{\mathrm{mg}}{\mathrm{mL}}\right)}\times 1000$$

### 2.6. High-Resolution Respirometry

High-resolution respirometry was conducted using 2 double-chambered Oxygraphy-2k machines (O2k, Oroboros Instruments, Innsbruck, Austria; https://www.oroboros.at accessed on 1 October 2023) according to a modified SUIT-008 protocol. Following equipment calibration as per manufacturer specs, chambers were filled with 2 mL of MiR05 (pre-analysis oxygen concentration between 195 and 205 nmol/mL) [32]. Homogenized hemispheric cortical and hippocampal tissue samples (100 µL ± 1%) were injected into O2k chambers via Hamilton syringe (Hamilton^®^ Gastight^®^ 1700 series; Sigma-Aldrich, St. Louis, Missouri, USA). Mitochondrial oxygen concentration (µM) and oxygen flux (pmol/(s·mL)) were recorded in real-time (DatLab 7 software, Oroboros Instruments, Austria) during induced respiratory states via polarographic measurement at 37 °C with electronic Peltier regulation. 

Use of a modified SUIT-008 protocol, with the presence of glycerophosphate to provide respiratory stimulation after complex I inhibition, followed an order of chemical addition that induced respiratory states progressively through the mitochondrial chain complexes. Full details of these chemical effects are found in our previous study [23]. Polarographic measurements were made during non-phosphorylating LEAK state to determine CI-linked and CI&II-linked OXPHOS capacities, CI&II-linked and CII-linked ET capacities, and residual oxygen consumption (ROX) after CIII inhibition. Electron transfer (ET) coupling efficiency, a value associated with complex-linked electron transport capacities, was calculated as follows:

$$ET\;coupling\;efficiency=\frac{1-respiratory\;rate\;of\;CI\&CII\;OXPHOS\;state}{respiratory\;rate\;of\;CI\&CII\;ET\;state}$$

### 2.7. Measurement of Mitochondrial H_2_O_2_ Flow Formation

Hydrogen peroxide production was measured in homogenized hemispheric cortical tissues using the O2k-Fluo LED2-Module combined with Fluorescence-Sensor Green (Oroboros Instruments, Austria) using a SUIT-009 AmR mt D021 protocol pattern (https://www.bioblast.at/index.php/SUIT-009_AmR_mt_D021 accessed on 5 November 2023). Full details of ROS production measurement can be found in our previous study [23]. Residual oxygen consumption, which reflects oxygen consumption from undefined sources, was measured at this time [33].

### 2.8. Statistical Analysis 

Statistical analysis and graphing were conducted in GraphPad Prism (Version 7.0; GraphPad Software, Inc., Boston, MA, USA). Data, with statistical significance set to *p* < 0.05, were assessed for normal distribution and presented as mean values ± standard error of the mean (S.E.M.). Comparisons within the same group (fMCAo or Sham) between cerebral hemispheres were assessed with a paired Student’s *t*-test. Comparisons between fMCAo and Sham groups were assessed with an unpaired *t*-test. One-way analysis of variance (ANOVA), followed by a post hoc Tukey’s multiple comparison test, was applied to oxygen and hydrogen peroxide flux data over time for ipsilateral and contralateral cerebral hemispheres of fMCAo and Sham mice. 

## 3. Results

### 3.1. Mitochondrial Oxygen Consumption

To investigate the long-term effects of ischemic brain damage on region-specific mitochondrial functioning in mice caused by 60 min of filament-induced middle cerebral artery occlusion (fMCAo), the mitochondrial respiration rates of cortical and hippocampal tissues were measured during OXPHOS- and ET-induced states at set time periods following fMCAo: 60, 90, 120, and 180 days after circulatory reperfusion. Respiratory measurements were compared within and between two study groups, fMCAo and Sham (quick, one-time filament-induced vascular wall irritation). Within each experimental study group, for each individual mouse, ipsilateral (ischemic) hemisphere data were compared to contralateral (non-ischemic) data at each defined time period. Additionally, the same hemisphere in each experimental group was compared over time. 

#### 3.1.1. Differences in Tissue Mass-Specific Oxygen Fluxes between Hemispheres in Cortical and Hippocampal Tissue in One Time Period

**Figure 1:**
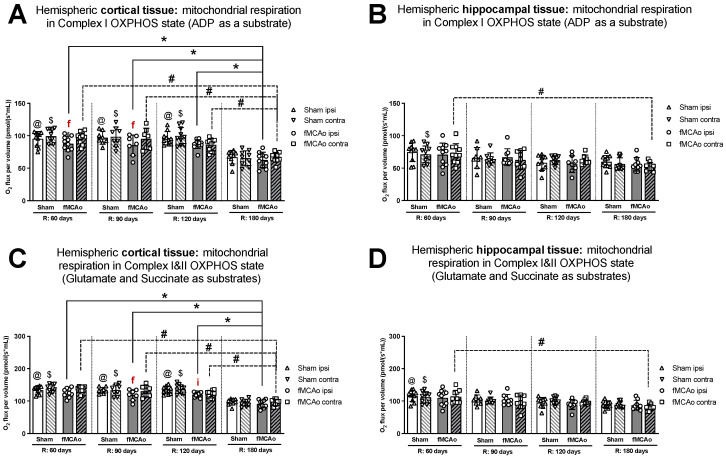
Long-term (60, 90, 120, and 180 days after reperfusion) mitochondrial oxygen consumption changes in (**A**) cortical tissue Complex I, (**B**) hippocampal tissue Complex I, and (**C**) cortical tissue Complexes I&II; (**D**) hippocampal tissue Complexes I&II, presented as oxygen flux per volume (pmol/(s*mL)), in induced OXPHOS states in mice. Mitochondrial respiration was measured using the Oroboros O2k-FluoRespirometer, where R denotes time elapsed since reperfusion, solid lines indicate comparisons between fMCAo ipsilateral hemispheres, and dotted lines indicate comparisons between fMCAo contralateral hemispheres. Statistical comparisons were made for each test group over time and between ipsilateral and contralateral hemispheres. Significant changes over time, determined by one-way ANOVA analysis, are denoted by * *p* < 0.05 vs. fMCAo ipsi, R: 180 days; ^#^ *p* < 0.05 vs. fMCAo contra, R: 180 days; ^@^ *p* < 0.05 vs. Sham ipsi, R: 180 days; and ^$^ *p* < 0.05 vs. Sham contra, R: 180 days. Significant changes between hemispheres at one time-point, determined by a paired, non-parametric *t*-test, are denoted by ^f^ *p* < 0.05 vs. fMCAo contra and ^i^ *p* < 0.05 vs. Sham ipsi. Data are presented as mean oxygen flux per volume ± SD. Sample size for all state respirometry measures of cortical tissue: Sham 60 days: n = 10, Sham 90 days: n = 8, Sham 120 days: n = 10, Sham 180 days: n = 9; fMCAo R: 60 days: n = 12, fMCAo R: 90 days: n = 7, fMCAo R: 120 days: n = 11, fMCAo R: 180 days: n = 10. Sample of hippocampal tissue: Sham 60 days: n = 10, Sham 90 days: n = 8, Sham 120 days: n = 9, Sham 180 days: n = 10; fMCAo R: 60 days: n = 12, fMCAo R: 90 days: n = 9, fMCAo R: 120 days: n = 8, fMCAo R: 180 days: n = 9.

**Figure 2:**
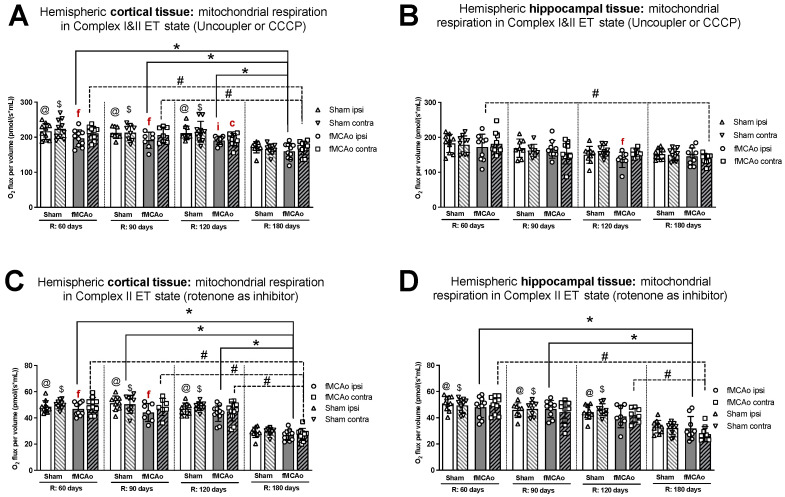
Long-term (60, 90, 120, and 180 days after reperfusion) mitochondrial oxygen consumption changes in (**A**) cortical tissue Complex I&II, (**B**) hippocampal tissue Complex I&II, and (**C**) cortical tissue Complex II, (**D**) hippocampal tissue Complex II, presented as oxygen flux per volume (pmol/(s*mL)), during induced ET states in mice. Mitochondrial respiration was tested by using the Oroboros O2k-FluoRespirometer, where R denotes time elapsed since reperfusion, solid lines indicate comparisons between fMCAo ipsilateral hemispheres, and dotted lines indicate comparisons between fMCAo contralateral hemispheres. Statistical comparisons were made for each test group over time and between ipsilateral and contralateral hemispheres. Significant changes over time, determined by one-way ANOVA analysis, are denoted by * *p* < 0.05 vs. fMCAo ipsi, R: 180 days; ^#^ *p* < 0.05 vs. fMCAo contra, R: 180 days; ^@^ *p* < 0.05 vs. Sham ipsi, R: 180 days; and ^$^ *p* < 0.05 vs. Sham contra, R: 180 days. Significant changes between hemispheres at one time point, determined by a paired, non-parametric *t*-test, are denoted by ^f^ *p* < 0.05 vs. fMCAo contra; ^i^ *p* < 0.05 vs. Sham ipsi; and ^c^ *p* < 0.05 vs. Sham contra. Data are presented as mean oxygen flux per volume ± SD. Sample size for all state respirometry measures of cortical tissue are as follows: Sham 60 days: n = 10, Sham 90 days: n = 8, Sham 120 days: n = 10, Sham 180 days: n = 9; fMCAo R: 60 days: n = 12, fMCAo R: 90 days: n = 7, fMCAo R: 120 days: n = 11, fMCAo R: 180 days: n = 10. Sample of hippocampal tissue: Sham 60 days: n = 10, Sham 90 days: n = 8, Sham 120 days: n = 9, Sham 180 days: n = 10; fMCAo R: 60 days: n = 12, fMCAo R: 90 days: n = 9, fMCAo R: 120 days: n = 8, fMCAo R: 180 days: n = 9.

Within ipsilateral and contralateral cortical tissue, disparities between hemispheres persisted several months post-ischemic stroke, wherein the ipsilateral hemisphere experienced lower rates of mitochondrial oxygen consumption. Significant differences between hemispheric cortical tissues were observed 60 days after fMCAo during CI OXPHOS (^f^ *p* = 0.0269; Figure 1A), CI&II ET (^f^ *p* = 0.0020l; Figure 2A), and CII ET (^f^ *p* = 0.0273; Figure 2C) states. Significant differences between hemispheres were also observed 90 days after fMCAo for all induced states: CI OXPHOS (^f^ *p* =0.0469; Figure 1A), CI&II OXPHOS (^f^ *p* = 0.0313; Figure 1C), CI&II ET (^f^ *p* = 0.0292; Figure 2A), and CII ET (^f^ *p* = 0.0241; Figure 2C).

Within hippocampal tissues of fMCAo group mice, significant differences were observed between hemispheres only during CI&II ET state 120 days (^f^ *p* = 0.0234; Figure 2B) after surgery, where respiration in the ipsilateral hemisphere was significantly lower. There were no statistically significant differences between hemispheres of Sham group mice in either cortical or hippocampal tissues (Figure 1 and Figure 2).

Significantly lower mitochondrial oxygen consumption in the fMCAo group ipsilateral hemisphere, compared to Sham group ipsilateral hemisphere data, was observed in cortical tissue samples during CI&II OXPHOS (^i^ *p* = 0.0096; Figure 1C) and CI&II ET (^i^ *p* = 0.0105; Figure 2A) samples 120 days after surgery. There were no statistically significant differences between fMCAo and Sham group ipsilateral hemisphere data in hippocampal tissue samples. When comparing contralateral hemisphere fMCAo group data with Sham group contralateral hemisphere data, no differences were denoted in both cortical and hippocampal tissues.

#### 3.1.2. Differences in Tissue Mass-Specific Oxygen Fluxes between Sham and fMCAo Groups in Hippocampal and Cortical Tissue

It is noteworthy that differences between Sham and fMCAo groups remained evident at 60 days, 90 days, and 120 days post-ischemic stroke/surgery. Comparing Sham and fMCAo ipsilateral hemisphere cortical tissues at each time point after surgery, day 120 mitochondrial oxygen consumption was significantly different during CI&II OXPHOS (^@^ *p* = 0.0125; Figure 1C) and CI&II ET (^@^ *p* = 0.0041; Figure 2A). Significant differences between Sham and fMCAo contralateral hemispheres were also observed on day 120 during CI&II OXPHOS (^$^ *p* = 0.0465; Figure 1C) and CI&II ET (^$^ *p* = 0.0465; Figure 2A). Within hippocampal tissues, no statistically significant differences between Sham and fMCAo groups were observed. 

#### 3.1.3. Differences in Tissue Mass-Specific Oxygen Fluxes in Cortical Tissue over Time

Following ANOVA analysis of oxygen fluxes in cortical tissue during induced respiratory states (Figure 1A,C and Figure 2A,C), both Stroke and Shame group ipsilateral and contralateral hemispheres presented with a significant decrease in respiration on day 180 compared to day 60, 90, and 120 measurements. Exceptions to this trend occurred in the Stroke group’s contralateral hemisphere on day 120 during CI&II ET (Figure 2A) and all Sham group contralateral hemisphere measurements during CI OXPHOS (Figure 1A). Table 1 contains a summary of *p*-values following statistical analysis for the Stroke and Sham group ipsilateral and contralateral hemispheres during induced respiratory states.

#### 3.1.4. Differences in Tissue Mass-Specific Oxygen Fluxes in Hippocampal Tissue over Time

After analysis of fMCAo ipsilateral hippocampal tissues, no significant differences in oxygen flux were observed over time for the following induced states: CI OXPHOS (Figure 1B), CI&II OXPHOS (Figure 1D), and CI&II ET (Figure 2B). A significant difference in oxygen flux was observed during CII ET, induced after the inhibition of CI with rotenone, where day 180 mitochondrial respiration was lower compared to day 60 and day 90 respiration (* *p* = 0.0014, * *p* = 0.0049, respectively; Figure 2D).

Regarding fMCAo contralateral hippocampal tissues, significantly lower rates of mitochondrial respiration were observed on day 180 compared to day 60 during CI OXPHOS (^#^ *p* = 0.0119; Figure 1B; F (4, 41) = 4019), during CI&II OXPHOS (^#^ *p* = 0.0023; Figure 1D), and during CI&CII ET (^#^ *p* = 0.0061; Figure 2B). Significant differences across all time periods were observed only during CII ET, where lower respiratory fluxes were recorded on day 180 compared to day 60, day 90, and day 120 respiratory fluxes (^#^ *p* < 0.0001, ^#^ *p* < 0.0001, ^#^ *p* = 0.0007, respectively; Figure 2D). 

Sham group respiration in contralateral hippocampal tissues on day 180 was significantly decreased compared to day 60 respiration during CI OXPHOS (^$^ *p* = 0.0238; Figure 1B), during CI&II OXPHOS (^$^ *p* = 0.0034; Figure 1D), and during CI&II ET (^$^ *p* = 0.0256; Figure 2B). During CII ET, after rotenone-induced inhibition, Sham contralateral hemisphere hippocampal oxygen flux on day 180 was significantly lower than day 60, day 90, and day 120 oxygen fluxes (^$^ *p* < 0.0001, all data points; Figure 2D).

#### 3.1.5. Differences in ET Coupling Efficiency between Hemispheres in Cortical and Hippocampal Tissue 

**Figure 3:**
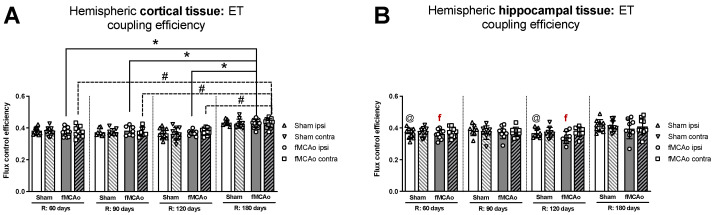
Long-term (60, 90, 120, and 180 days after reperfusion) (**A**) cortical and (**B**) hippocampal mitochondrial ET coupling efficiencies, or uncoupler effect, with internal normalizations (expressing respiratory flux after addition of ETS uncoupler relative to respiratory flux with pyruvate, malate, ADP, glutamate, and succinate). Mitochondrial coupling efficiencies were calculated based on oxygen flux data obtained with Oroboros O2k-FluoRespirometer, where R denotes time elapsed since reperfusion, solid lines indicate comparisons between fMCAo ipsilateral hemispheres, and dotted lines indicate comparisons between fMCAo contralateral hemispheres. Statistical comparisons were made for each test group over time and between ipsilateral and contralateral hemispheres. Significant changes over time, determined by one-way ANOVA analysis, are denoted by * *p* < 0.05 vs. fMCAo ipsi, R: 180 days; ^#^ *p* < 0.05 vs. fMCAo contra, R: 180 days; and ^@^ *p* < 0.05 vs. Sham ipsi, R: 180 days. Significant changes between hemispheres at one time point, determined by a paired, non-parametric *t*-test, are denoted by ^f^ *p* < 0.05 vs. fMCAo contra. Data, corrected for background noise and residual oxygen consumption, are presented as flux control ratio ± SD. Sample size: Sham 60 days: n = 10, Sham 90 days: n = 8, Sham 120 days: n = 10, Sham 180 days: n = 9; fMCAo R: 60 days: n = 12, fMCAo R: 90 days: n = 7, fMCAo R: 120 days: n = 11, fMCAo R: 180 days: n = 10. Sample of hippocampal tissue: Sham 60 days: n = 10, Sham 90 days: n = 8, Sham 120 days: n = 9, Sham 180 days: n = 10; fMCAo R: 60 days: n = 12, fMCAo R: 90 days: n = 9, fMCAo R: 120 days: n = 8, fMCAo R: 180 days: n = 9.

No significant differences in ET coupling efficiency measurements, known as an uncoupler effect and presented as flux control efficiency, were observed between hemispheres in either fMCAo or Sham cortical tissues (Figure 3A). Significant differences in ET coupling efficiency measurements were observed between hemispheres in fMCAo hippocampal tissues on day 60 and day 120 (^f^ *p* = 0.0488 and ^f^ *p* = 0.0078, respectively; Figure 3B), where ipsilateral hippocampal flux control rates were lower than contralateral rates. There were no significant differences between hemispheres for the Sham group when hippocampal ET coupling efficiency was assessed (Figure 3B).

#### 3.1.6. Differences in ET Coupling Efficiency in Cortical and Hippocampal Tissue over Time

Analysing ET coupling efficiency in cortical tissue samples over time, the highest flux control efficiency was observed on day 180 in both fMCAo hemispheres (Figure 3A). In the fMCAo ipsilateral hemisphere, flux control efficiency was significantly higher on day 180 compared to day 60, day 90, and day 120 efficiency (* *p* < 0.0001, * *p* = 0.0007, * *p* = 0.0001, respectively; Figure 3A). In the fMCAo contralateral hemisphere, flux control efficiency was also significantly higher on day 180 compared to day 60, day 90, and day 120 ratios (^#^ *p* < 0.0001, all data points; Figure 3A). There were no significant differences in ET coupling efficiency in cortical tissues over time for either Sham hemisphere (Figure 3A). 

After measuring ET coupling efficiency in hippocampal tissues, no significant differences in flux control efficiency over time were observed in either hemisphere for both fMCAo and Sham groups (Figure 3B). 

#### 3.1.7. Representative Mitochondrial Measurements following Substrate Additions

**Figure 4:**
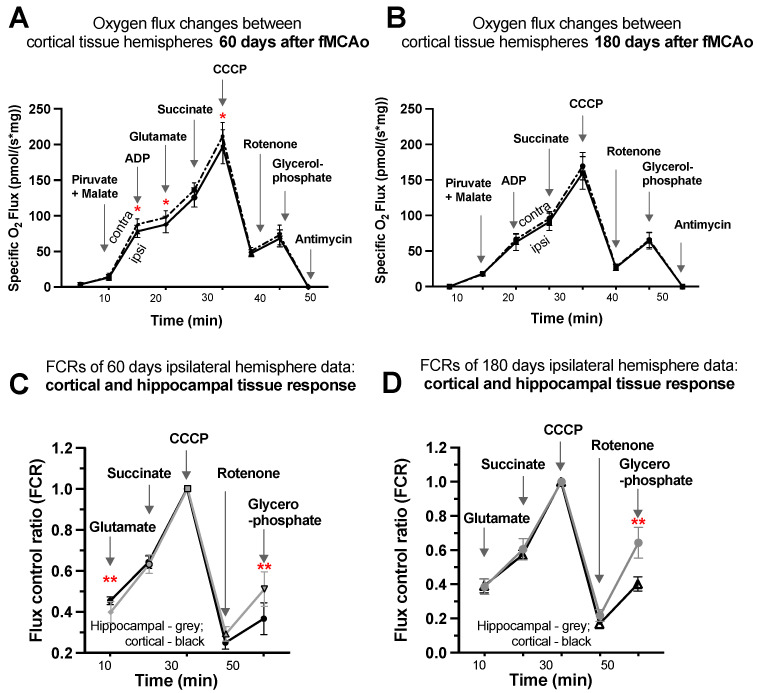
Graphical representations of background-corrected (specific) oxygen flux changes between cortical tissue hemispheres of fMCAo group mice (**A**) 60 days and (**B**) 180 days following reperfusion and standardized flux control ratio (FCRs), normalized for maximum flux, of cortical and hippocampal tissue (**C**) 60 days and (**D**) 180 days after reperfusion, where solid black lines indicate cortical ipsilateral oxygen flux (**A**,**B**) or cortical FCRs (**C**,**D**), dotted lines indicate cortical contralateral oxygen flux (**A**,**B**), and gray lines indicate hippocampal ipsilateral FCRs (**C**,**D**). Analyzed by unpaired *t*-test, significant changes are denoted by * *p* < 0.05 vs. fMCAo ipsi cortical tissue and ** *p* < 0.05 vs. fMCAo ipsi hippocampal tissue. Sample size of cortical tissue measurements: Sham 60 days: n = 10, fMCAo R: 60 days: n = 12, Sham 180 days: n = 9; fMCAo R: 180 days: n = 10. Sample size of hippocampal tissue measurements: Sham 60 days: n = 10, Sham 180 days: n = 10; fMCAo R: 60 days: n = 12, fMCAo R: 180 days: n = 9.

Four representative graphs are provided using oxygen flux measurements (Figure 4A,B) and control factor ratios (Figure 4C,D) taken on day 60 and day 180 following surgery. Statistical evaluations and interpretations for representative graphs correspond to the results provided in Figure 1 and Figure 2. Within these graphs, oxygen flux and control factor ratio changes can be tracked following chemical additions, indicating respiration state induction, with global changes and shifts in mitochondrial function easily observed.

Considering oxygen flux measurements 60 days after surgery (Figure 4A), significantly lower oxygen consumption was observed following additions of ADP, glutamate, and CCCP (denoted by *). No significant changes were observed following additions of pyruvate/malate, succinate, rotenone, glycerophosphate, or antimycin A, as well as after any substrates 180 days after surgery (Figure 4B).

To obtain factor control ratios (Figure 4C,D), oxygen flux data were standardized for maximal mitochondrial capacity after the addition of an uncoupler (CCCP), allowing for tissue response comparisons without concern for cell density variability in samples. Visible and significant differences were observed between cortical and hippocampal tissue after the addition of glutamate (during CI OXPHOS) 60 days after fMCAo (** *p* = 0.0053; Figure 4C) and after the addition of glycerophosphate (CII ET) 60 days after fMCAo (** *p* = 0.0017; Figure 4C). The glycerophosphate response in hippocampal tissue was greater 180 days after fMCAo (** *p* < 0.0001; Figure 4D).

### 3.2. Mitochondrial ROS Production in Cortical Tissues

Mitochondrial ROS production, in the form of H_2_O_2_ emissions, was measured in the cortical tissues of mice during CII-supported states at the following time periods after fMCAo and/or circulatory reperfusion: 60, 90, 120, and 180 days. Hippocampal tissues were not assessed for mitochondrial ROS production due to inadequate tissue amounts that were below testing standards and would have produced unreliable, nonconclusive data output.

#### 3.2.1. Differences in Tissue Mass-Specific H_2_O_2_ Emissions between fMCAo Cortical Hemispheres 

**Figure 5:**
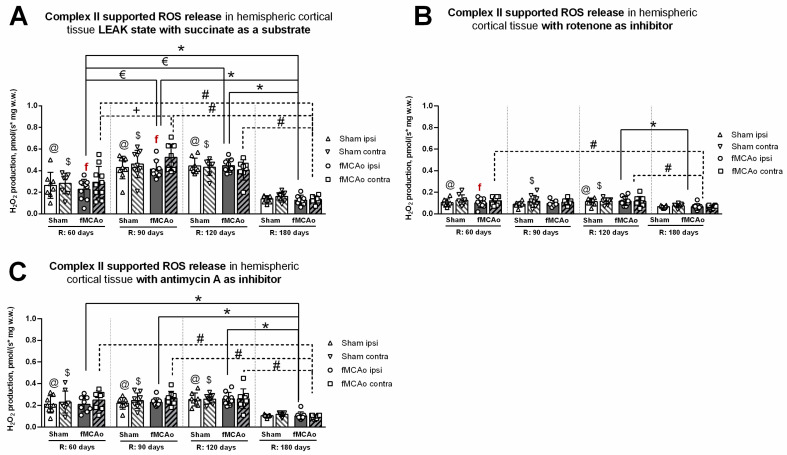
Long-term (60, 90, 120, and 180 days after reperfusion) cortical ROS emissions, presented as H_2_O_2_ production normalized by tissue mass (pmol/(s*mg w.w.)), during Complex II supported states in mice (**A**) in cortical tissue LEAK state, (**B**) in cortical tissue after inhibition of Complex I with rotenone, and (**C**) in cortical tissue after inhibition of Complex III with Antimycin A. Cortical ROS emissions in mitochondria were measured using the Oroboros O2k-FluoRespirometer, where R denotes time elapsed since reperfusion, solid lines indicate comparisons between fMCAo ipsilateral hemispheres, and dotted lines indicate comparisons between fMCAo contralateral hemispheres. Statistical comparisons were made for each test group over time and between ipsilateral and contralateral hemispheres. Significant changes over time, determined by one-way ANOVA analysis, are denoted by * *p* < 0.05 vs. fMCAo ipsi, R: 180 days; ^€^ *p* < 0.05 vs. fMCAo ipsi, R: 60 days; ^#^ *p* < 0.05 vs. fMCAo contra, R: 180 days; ^+^ *p* < 0.05 vs. fMCAo contra, R: 60 days; ^@^ *p* < 0.05 vs. Sham ipsi, R: 180 days; and ^$^ *p* < 0.05 vs. Sham, contra R: 180 days. Significant changes between hemispheres at one time point, determined by a paired, non-parametric *t*-test, are denoted by ^f^ *p* < 0.05 vs. fMCAo contra. Data are presented as H_2_O_2_ production normalized by mass (pmol/(s*mg w.w.)) ± SD. Sample size for H_2_O_2_ measurements: Sham 60 days: n = 9, Sham 90 days: n = 10, Sham 120 days: n = 9, Sham 180 days: n = 10; fMCAo R: 60 days: n = 10, fMCAo R: 90 days: n = 9, fMCAo R: 120 days: n = 9, fMCAo R: 180 days: n = 12.

Differences in H_2_O_2_ emissions were examined between cortical hemispheres within each time period. ROS release during induced CII-supported LEAK state (succinate as a substrate; Figure 5A) was significantly higher in the fMCAo contralateral hemisphere compared to the ipsilateral hemisphere on day 60 and day 90 (^f^ *p* = 0.0324 and ^f^ *p* = 0.0343, respectively; Figure 5A). No significant differences were noted between fMCAo hemispheres on day 120 or day 180. 

After inhibiting CI with rotenone (Figure 5B), overall ROS emission decreased in all test groups. However, a statistically significant difference was only observed on day 60, where ROS release was higher in the fMCAo group contralateral hemisphere compared to the ipsilateral hemisphere (^f^ *p* = 0.0280; Figure 5B). There were no significant differences in ROS emissions between fMCAo cortical hemispheres after the addition of antimycin A to cortical tissue samples (Figure 5C).

#### 3.2.2. Differences in Tissue Mass-Specific H_2_O_2_ Emissions in Cortical Hemispheres over Time

Considering that CI&II supported ROS emissions, significant time-related changes were observed in the fMCAo ipsilateral hemisphere (Figure 5A). ROS release was lowest in the fMCAo ipsilateral hemisphere on day 180 and significantly lower than on day 60, day 90, and day 120 (* *p* = 0.0403, * *p* < 0.0001, * *p* < 0.0001, respectively; Figure 5A). Significantly lower ROS release was also observed on day 60 in the fMCAo ipsilateral hemisphere compared to day 90 and day 120 (*^€^ p* < 0.0001; Figure 5A). Analysis of fMCAo contralateral hemisphere ROS emissions over time (Figure 5A) indicated a significant increase in H_2_O_2_ production from day 60 after surgery to day 90 (^+^ *p*=0.0007; Figure 5A). H_2_O_2_ production decreased to a minimum on day 180, significantly so when compared to day 60, day 90, and day 120 (^#^ *p* = 0.0224, ^#^ *p* < 0.0001, ^#^ *p* = 0.0001, respectively; Figure 5A) within the fMCAo contralateral hemisphere. During this succinate-supported state, Sham ipsilateral hemisphere ROS emissions were lowest on day 180 and significantly different compared to day 60, day 90, and day 120 (^@^ *p* = 0.0316, ^@^ *p* < 0.0001, ^@^ *p* < 0.0001, respectively; Figure 5A). In the Sham contralateral hemisphere, day 180 ROS emissions were again lowest and significantly different compared to day 60, day 90, and day 120 (^$^ *p* < 0.0460, ^$^ *p* < 0.0001, ^$^ *p* < 0.0001, respectively; Figure 5A).

Analyzing ROS emissions following rotenone addition, H_2_O_2_ production was significantly lower on day 180 in the fMCAo ipsilateral hemisphere compared to day 120 (* *p* = 0.0246; Figure 5B). Within the fMCAo contralateral hemisphere, emissions were lowest on day 180, with significant differences in H_2_O_2_ production compared to day 60 and day 120 (^#^ *p* = 0.0065 and ^#^ *p* = 0.0152; Figure 5B). Similar significant differences in results were observed for Sham ROS emissions during the rotenone-induced state. Compared to the lowest ROS emissions on day 180, day 60, and day 120, ROS emissions were significantly higher in the ipsilateral hemisphere (^@^ *p* = 0.0277 and *^@^ p* = 0.0051; Figure 5B) and in the contralateral hemisphere (^$^ *p* < 0.0277 and ^$^ *p* < 0.0051; Figure 5B).

After adding antimycin A, an ETC inhibitor and ROX-state enabler, a global decrease in mitochondrial respiration, measured as H_2_O_2_ production, was observed for both test groups—Sham and fMCAo—and both hemispheres over the entire test period. In the fMCAo ipsilateral hemisphere, day 180 ROS emissions were significantly lower compared to day 60, day 90, and day 120 (* *p* = 0.0023, * *p* = 0.0003, * *p* < 0.0001, respectively, Figure 5C). In the fMCAo contralateral hemisphere, ROS emissions exhibited a similar trend, wherein significantly lower H_2_O_2_ production occurred on day 180 compared to day 60, day 90, and day 120 (^#^ *p* = 0.0010, ^#^ *p* = 0.0003, ^#^ *p* = 0.0003, respectively; Figure 5C). H_2_O_2_ production in Sham hemispheres followed the same trend, where day 180 ROS emissions were significantly lower compared to day 60, day 90, and day 120 in the ipsilateral hemisphere (^@^ *p* = 0.0032, ^@^ *p* = 0.0012, ^@^ *p* < 0.0001, respectively; Figure 5C) and in the contralateral hemisphere (^$^ *p* < 0.0057, ^$^ *p* = 0.0018, ^$^ *p* = 0.0006, respectively; Figure 5C).

### 3.3. Representation of Infarct Size following a 60 min Long fMCAo

**Figure 6:**
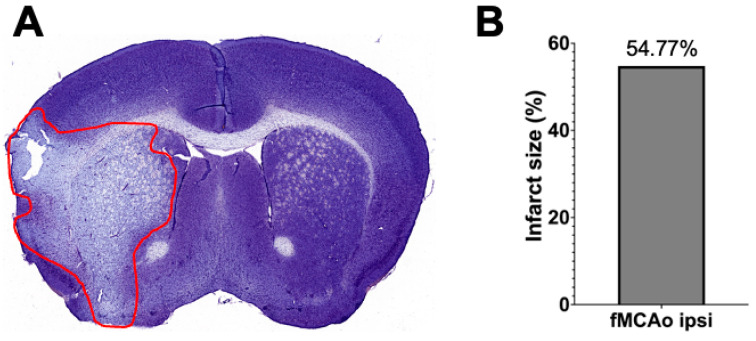
Infarct volume assessment is illustrated by (**A**) a representative photograph of a Nissl-stained coronal brain section (the red line states the perimeter of the ischemic zone) in a 60 min long fMCAo mouse model (2× microscope’s magnification) and (**B**) the graph illustrates the infarct area of the ischemic hemisphere expressed in infarct area percentage of whole hemisphere area.

Infarct volume assessment was made using the Nissl staining technique (Figure 6A) performed 24 h after I/R damage in a 60 min long fMCAo model in C57BL/6 mouse. Infarct size, represented as infarct area, was calculated as a percentage of the ischemic area of the total hemisphere area. The percentage of the representative ischemic hemisphere’s infarct area was 54.77% (Figure 6B). 

## 4. Discussion

Ischemia/reperfusion injury describes secondary injury that occurs after reperfusion and is linked to the formation of ROS and tissue damage. So far, early consequences of ischemic stroke have been mainly studied by comparing the pathological alterations in the ipsilateral hemispheres of stroke animals with those of sham animals only or by analyzing whole brain samples taken from stroke and sham animals [34,35]. Recently, by using high-resolution fluorespirometry, we demonstrated that cortical tissue from the ipsilateral hemisphere had lower mitochondrial oxygen consumption in Complex I- and II-linked respiration during OXPHOS and ET states and higher ROS production compared to the contralateral (non-ischemic) hemisphere in mice directly after a 60 min long fMCAo and 2 h after reperfusion that corrected to near baseline 72 h after reperfusion. Thus, our previous data indicated a pattern of ischemia/reperfusion damage with a pronounced difference between the ipsilateral and contralateral hemispheres [23]. 

There is, however, a lack of knowledge on the long-term consequences of ischemic stroke and subsequent ischemia/reperfusion injury on the activity of the mitochondrial respiratory complexes with independent analysis of each cerebral hemisphere. To gain a comprehensive understanding of the entire brain response and processes involved in ischemia/reperfusion, both hemispheres must be examined independently. 

Thus, the present study offers insights into the long-term impact of ischemia/reperfusion-induced injury on mice brain mitochondrial bioenergetics by including several important aspects: (a) the use of the highly sensitive Oxygraph-2k and high-resolution fluorespirometry to examine mitochondrial electron transport through the respiratory complexes via oxygen consumption (in pmol) and levels of reactive oxygen species via H_2_O_2._ emission; (b) comparison of mitochondria functioning in both hemispheres (ipsi- or *ischemic* vs. contralateral or non-*ischemic*); (c) examination of region-specific (cortical and hippocampal) tissues; (d) and a long-term study design with respirometry measurements occurring 60, 90, 120, and 180 days after a 60 min long fMCAo followed by reperfusion. Given the evidence that high-resolution respirometry performed on isolated mitochondria can lead to artificial mitochondria membrane damage, a gentle but reliable method of tissue homogenization with a glass Dounce homogenizer was used in this study to preserve mitochondrial integrity [36,37]. This optimized preparation of brain tissue yields a high degree of permeabilization, allowing respiratory state induction, as was evident by the minimal effect digitonin titrations had on OXPHOS capacity [36]. Following a substrate-induced respirometry technique with the Oxygraph-2k allowed us to accurately collect data and detect subtle changes in mitochondrial bioenergetics at complexes I and II of the electron transport system and during each respiratory state [38]. 

Our findings show that even 120 days after 60 min of cerebral ischemia, the ipsilateral cortical hemisphere respiration was significantly lower in fMCAo group mice compared to the Sham group during CI&II OXPHOS (succinate as a substrate; Figure 1C) and CI&II ET (maximal oxygen consumption following electron transport system uncoupling; Figure 2A). This indicates that considerable long-term alterations in mitochondrial bioenergetics caused by stroke can be present in the ischemic hemisphere of fMCAo mice several months after the event. In a rat study 30 days after tMCAo, one observed pathological feature was extensive vascular damage, such as BBB impairment with damage mostly seen in the ipsilateral striatum and motor cortex; furthermore, microvascular alterations were observed in ultrastructural level contralateral cerebral brain structures [25].

We found that the long-term effects of ischemic stroke on cortical tissue include significantly lower mitochondrial oxygen consumption in the ischemic (ipsilateral) hemisphere compared to the non-*ischemic* (contralateral) hemisphere on day 60 after fMCAo in CI OXPHOS (ADP and glutamate as substrates) and on day 90 during all CI&II respiratory states (Figure 1A,C and Figure 2A,C). Conversely, the same respiratory state measurements taken 120 and 180 days after stroke revealed no longer significant differences in oxygen consumption between cortical hemispheres. 

Further, we demonstrated in the CI&II ET state that following the addition of a respiratory system uncoupler, absolute flux control measurements taken over a 120-day period after ischemia/reperfusion revealed significantly lower oxygen fluxes in the ipsilateral hemisphere of hippocampus tissue compared to the contralateral hemisphere (Figure 2B). In a short-term study by Butler and co-authors, it was described that neuronal degeneration in the hippocampus closer to the infarct region tends to occur between 12 h and 7 days after injury, with neuronal death reaching a peak at 4 days [39]. In a mouse model, in situ hybridization study of the hippocampus up to 1 month after MCAo indicated no difference in gene expression between ipsilateral and contralateral hemispheres for several metabolism, protein folding, and signal transduction genes [40]. Other research using a rat model showed that hippocampal oxygen consumption 2, 3, and 4 months post-ischemia (via permanent bilateral occlusion of the common carotid arteries) was significantly decreased when compared with month-matched pseudo-operation groups [41]. A long-term human stroke study noted that ten years after a stroke event, generalized and injurious effects were observed in ipsilateral brain structures remote from the patient infarct site, such as the ipsilateral hippocampus and thalamus, due to infarct expansion and secondary neuronal damage [42]. 

Flux control efficiencies, which are independent of mitochondrial density, can be used to assess qualitative changes in OXPHOS patterns and provide additional information on altered OXPHOS regulation. ET coupling efficiency as an uncoupler effect expresses the impact of CCCP/uncoupler as a fractional change of oxygen flux, normalized to a reference state—pyruvate, malate, adenosine diphosphate, glutamate, and succinate coupled respiration [38]. As noted previously, flux control efficiencies provide standardized substrate values that eliminate possible fresh weight measurement inaccuracies and bolster findings observed within oxygen flux data [36]. Following uncoupler addition, our data analysis revealed significantly lower ET coupling efficiency in the ipsilateral than in the contralateral hemisphere of hippocampal tissue 60 and 120 days after ischemic reperfusion injury (Figure 3B). 

Additional research has demonstrated the accumulation of succinate during ischemia [12]. Further to this, succinate undergoes rapid oxidation by succinate dehydrogenase (SDH) after blood flow reperfusion; in this way, SDH drives ROS production due to reverse electron transport from mitochondrial CII to CI [22,43]. The impact is such that succinate is considered a primary driver of mitochondrial ROS production within 5 min following reperfusion and can be attributed to underlying ischemia/reperfusion injury in a range of tissues, including the brain and heart [12]. Given that succinate-dependent H_2_O_2_ production is controlled by the pH gradient across the inner mitochondrial membrane and is depressed when the gradient is abolished, a decrease in succinate-dependent H_2_O_2_ production can serve as an indicator of compromised mitochondrial membrane potential (mtMP)—studies note even small (10%) reductions in mtMP can result in an up to 90% reduction in succinate-supported ROS production [44]. Our research revealed that the fMCAo group ipsilateral hemisphere had a significant decrease compared to the contralateral hemisphere 90 days after reperfusion in succinate-supported oxygen flux (CI&II OXPHOS state; Figure 1C) and 60 and 90 days after reperfusion in succinate-dependent H_2_O_2_ production (Figure 5A). These results suggest a change in balance between CI- and CII-dependent mitochondrial processes, as lower mitochondrial respiration was evident during respiratory states associated with both complexes. 

Given that differential functions are governed by specific brain regions, one could hypothesize that energy demand and bioenergetics also differ in how they operate between said brain regions. The transformation of absolute oxygen flux values into flux control ratios (FCRs), normalized to 1.0 (maximum flux as a common reference state), allows comparisons between cortical and hippocampal tissue samples with variable cell densities [38]. Following data analysis, we observed that cortical and hippocampal tissue responded differently to OXPHOS substrates (Figure 4C,D). After data normalization for the maximum flux of cortical and hippocampal tissue, ipsilateral hemisphere flux control ratios were calculated. Data analysis revealed a higher response to glutamate for cortical tissue 60 days after ischemia/reperfusion (Figure 4C). Ferrari et al. implied that different effects on brain mitochondria might be connected to the role of glutamate in boosting synaptic neuroplasticity after the acute phase of brain ischemia and upon recirculation. During post-ischemic recovery of complete 15 min long MCAo rats, somatic mitochondria did not show modifications of enzyme assay results, while in intra-synaptic mitochondria of adult animals, glutamate catabolism remained decreased up to 96 h in aged (2-year old) rats [45]. Published data indicate that the expression of plasma membrane glutamate transporters in the hippocampus CA1 area, including glutamate transporter 1 (GLT-1), increased 12 h after ischemia–reperfusion of MCA. A following downregulation of GLT-1 occurs at transcriptional and protein synthesis levels and persists 24 h after reperfusion. Downregulation, and thus a decline in GLT-1 generation, impairs normal clearance of synaptically released glutamate and may contribute to neural damage following a focal ischemic injury [35,46].

Further data review also indicated that tissue response to glycerol-3-phosphate varied between hippocampal and cortical tissues, with the hippocampus experiencing significantly increased glycerophosphate-mediated respiration in both 60 days and 180 days after ischemia/reperfusion (Figure 4C,D). Glycerol-3-phosphate, a redox substrate of mitochondrial glycerol-3-phosphate dehydrogenase, passes electrons to quinone, which then feeds them to complexes III and IV [33,47]. Some studies indicate that glycerol-3-phosphate increased immediately after global brain ischemia in frozen cerebral tissues [48]. There are currently no peer-reviewed studies on long-term glycerophosphate brain tissue response after ischemia/reperfusion. 

When comparing day 60 to day 90, 120, and 180 data, distinctions in activity between cerebral cortex hemispheres are most noticeable during respiration paired with the CI OXPHOS state (Figure 1A) and after inhibition of CI with rotenone (Figure 2C). We provided a summary of hemispheric differences in cortical tissue 60 days after ischemia/reperfusion. These data indicated that mitochondrial oxygen flux was lower in the ipsilateral hemisphere than in the contralateral after additions of ADP, glutamate, and uncoupler (CCCP) (Figure 4A), and no further differences were noted 180 days (6 months) after ischemia/reperfusion (Figure 4B). 

Several notable differences in response to ischemia/reperfusion injury between male and female mouse brains have dictated the decision to exclusively start by using male mice in this study. When compared to the male mouse response, female mice exhibit lower levels of oxidative stress and increased mitochondrial respiration following ischemia [49]. There was a smaller decrease in female brain ATP production after ischemia/reperfusion compared to the male brain, which could result in better endogenous protection following a stroke event [49]. Other studies show that stimulated and uncoupled respirations linked with flavin adenine dinucleotide (FADH_2_) were decreased specifically in female mice after a 60 min long MCAo that can be attributed to downregulation of activity at CII [50]. Other studies report that mitochondria from healthy male brains, compared to females, experienced significantly higher rates of H_2_O_2_ production during all resting and active respiratory states linked with NAD+ [51]. The results and conclusions from these studies imply that CI is more tightly coupled in female brain mitochondria and is a likely source of ROS and electron leakage of the mitochondrial electron transport system in male brains. Further to this, when in the presence of succinate and during respiration linked to NAD+, male-specific increases in ROS production were mostly abolished, suggesting that female mouse brains experience more ROS leakage at CII [51].

One factor to consider when interpreting the results and applying new knowledge is the impact that age will have on energy demand and bioenergetics across brain regions. Aging is reported to be linked with declining mitochondrial function and antioxidant detoxification, which leads to degradation and oxidative damage [52,53]. When examining our oxygen consumption data, a greater aging influence was observed in cortical rather than hippocampal tissues in mice (Figure 2A,B). In the context of our study, all mice were 2 months old at the time of ischemia/reperfusion. Accordingly, mouse ages during mitochondrial measurements were 4 months on R:60 (young adult), 5 months on R:90, 6 months on R:120, and 8 months on R:180 (middle age). Interpreting processes that are measured up to 6 months after ischemia/reperfusion injury, detrimental aging processes could be an additional contributor to the observed tissue damage. Our results were consistent with age effects, wherein significantly lower oxygen consumption was observed for fMCAo mice 6 months after ischemic stroke compared to consumption at other studied time periods (Figure 1A,C and Figure 2A,C, Table 1). Mitochondrial respiration in the fMCAo ipsilateral hemisphere during state 3 or maximal respiration was lowered in both cortical and hippocampal tissues. 

According to a study of aging-dependent impacts on mitochondria function within cortical tissue synaptosomes containing mitochondria only from neuronal origin, 17-month-old male mice showed decreases in respiration-driven proton leak and mitochondrial basal respiration of 26% and 33%, respectively, while spare respiratory capacity remained unaffected [54]. Respiratory control remained unaffected in non-synaptic cortical glia and astrocyte mitochondrial fractions from 17-month-old mice, yet compared with 3-month-young animals’ succinate-supported state, three respiratory rates were reduced by 45% [54]. Another study showed that synaptic mitochondria from the hippocampus of 12-month mice presented with premature bioenergetic dysfunction, as evidenced by an increase in ROS production and reduced ATP formation—an effect that does not occur in non-synaptic mitochondria, demonstrating that hippocampal synaptic mitochondria fail before non-synaptic in middle-aged mice [19]. A study using young, adult, middle-aged, and aged Brown Norway rats (1, 4, 12, and 24 months old, respectively) analyzed mitochondrial bioenergetics in several brain regions (brain stem, frontal cortex, hippocampus, cerebellum, and striatum) and found that the hippocampus experienced an age-related decline in ATP synthesis (State 3 respiration); activity of mitochondrial pyruvate dehydrogenase complex; enzyme activity of electron transport chain complexes I, II, and IV; and maximal respiratory capacities [55]. Other research using spectrophotometric methods compared mitochondrial enzyme activities and oxidative damage in ipsilateral/contralateral hemispheres, striatum, and cortex up to 7 days after a 60 min long tMCAo in young, young adult, middle-aged, and aged mice (1, 4, 11–12, and 18–19 months old, respectively), and regional subanalyses indicated that pro-oxidant/antioxidant imbalance occurred more prominently in subcortical areas [8]. Regarding Sham surgery outcomes, 48 h after the surgical event, there were modest effects on mitochondrial oxygen consumption rates in mice and substantial effects on Ca^2+^ spark activity, indicating that even minimal surgery and/or anesthesia have prolonged effects on mitochondrial function in cerebral arteries [24]. Our study shows that Sham group mice also had significantly lower oxygen consumption during the 6-month period. We can then hypothesize that mitochondrial protective mechanisms exist, as oxidative damages were mainly observed 3 to 4 months after surgery/vascular wall irritation. This study has certain limitations, such as a lack of information on the long-term impacts of ischemia/reperfusion on mitochondrial membrane potential and calcium signaling in both cerebral hemispheres of male and female mice. 

## 5. Conclusions

A knowledge gap on the long-term impact of ischemic stroke on mitochondrial respiration processes and ROS production in the brain was addressed through this study. We report significant hemispheric differences between mitochondrial bioenergetic profiles in the brains of male ischemic stroke model mice up to 120 days after ischemia/reperfusion. A deeper comprehension of processes initiated by blood flow reperfusion is essential to address disease response, improve long-term management, and ultimately provide better recovery outcomes. Monitoring mitochondrial functionality over an extended period, such as 6 months, allows analysts to comprehensively evaluate mitochondrial recovery and would help to identify the timeframes during which significant changes in mitochondrial function occur. 

## Figures and Tables

**Table 1:** Summary of cortical tissue oxygen flux ANOVA analysis *p*-values.

Treatment Group	Hemisphere	Measurement Day (R)	Induced Respiratory State			
			CI OXPHOS (Figure 1A)	CI&II OXPHOS (Figure 1C)	CI&II ET (Figure 2A)	CII ET (Figure 2C)
Stroke Group	Ipsilateral	60	* *p* < 0.0001	* *p* < 0.0001	* *p* = 0.0044	* *p* < 0.0001
		90	* *p* = 0.0037	* *p* = 0.0018	* *p* = 0.0110	* *p* < 0.0001
		120	* *p* = 0.0003	* *p* = 0.0001	* *p* = 0.0214	* *p* < 0.0001
	Contralateral	60	^#^ *p* = 0.0002	^#^*p* < 0.0001	^#^*p* = 0.0003	^#^ *p* < 0.0001
		90	^#^ *p* = 0.0023	^#^ *p* = 0.0010	^#^ *p* = 0.0206	^#^ *p* < 0.0001
		120	^#^ *p* = 0.0306	^#^ *p* = 0.0116	-	^#^ *p* < 0.0001
Sham Group	Ipsilateral	60	^@^ *p* < 0.0001	^@^ *p* < 0.0001	^@^ *p* = 0.0006	^@^ *p* < 0.0001
		90	^@^ *p* < 0.0001	^@^ *p* < 0.0001	^@^ *p* = 0.0216	^@^ *p* < 0.0001
		120	^@^ *p* < 0.0001	^@^ *p* < 0.0001	^@^ *p* = 0.0046	^@^ *p* < 0.0001
	Contralateral	60	-	^$^ *p* < 0.0001	^$^ *p* < 0.0001	^$^ *p* < 0.0001
		90	-	^$^ *p* < 0.0001	^$^ *p* = 0.0391	^$^ *p* < 0.0001
		120	-	^$^ *p* < 0.0001	^$^ *p* = 0.0012	^$^ *p* < 0.0001

A summary of *p*-values, with non-significance denoted by marked-out cells, following statistical ANOVA analysis of cortical tissue oxygen flux for Stroke and Sham group ipsilateral and contralateral hemispheres during induced respiratory states. Table values correspond to significances indicated in Figure 1A,C and Figure 2A,C. Significant changes over time, determined by one-way ANOVA analysis, are denoted by * *p* < 0.05 vs. fMCAo ipsi, R: 180 days; ^#^ *p* < 0.05 vs. fMCAo contra, R: 180 days; ^@^ *p* < 0.05 vs. Sham ipsi, R: 180 days; and ^$^ *p* < 0.05 vs. Sham contra, R: 180 days.

## Data Availability

Data available on request due to legal restrictions.
